# Supplementary material for: India consists of multiple food systems with scoioeconomic and environmental variations
Source: PLoS One. 2022 Aug 26;17(8):e0270342. doi: 10.1371/journal.pone.0270342 (PMC9416984; doi:10.1371/journal.pone.0270342)

India consists of multiple food systems with scoioeconomic and environmental variations

S1 Appendix

S1 Equation.

$$C_a = C \times \frac{M_h + M_f}{M_h + M_g}$$

Here, C_a denotes the adjusted calorie intake of the household. C represents the estimated calorie intake based on the HCES data. M_h is a number of meals consumed by the household members in the house or received through purchase or as assistance or payment (excluding meals received from other households). M_g denotes the number of meals consumed by non-members (e.g., guests, employees). M_f represents the number of meals received free from other households.

S1 Table. Consumer Unit.

Sex	No. of Consumer units assigned											
	Age in completed years											
	<1	1-3	4-6	7-9	10-12	13-15	16-19	20-39	40-49	50-59	60-69	70 +
Male	0.43	0.54	0.72	0.87	1.03	0.97	1.02	1.00	0.95	0.90	0.80	0.70
Female	0.43	0.54	0.72	0.87	0.93	0.80	0.75	0.71	0.68	0.64	0.51	0.50

S2 Table. Food Groups.

Food Groups	Food items in the data
Cereals	Rice – PDS, Rice – other sources, Chira, Khoi, Kawa, Muri and other Rice products, Wheat/atta – PDS, Wheat/atta – other sources, Maida, Suji, Rawa, Sewai, Noodles, Bread (bakery) and other Wheat products, Jowar & Products, Bajra & Products, Maize & Products, Barley & Products, Small Millets & Products, Ragi & Products, Other Cereals
White Tubers and Roots	Cereal substitutes (Tapioca, Jackfruit seed, etc., Potato (includes Sweet Potato and Green Plantain)
Vegetables	Onion, Tomato, Brinjal, Radish, Carrot, Palak/Other leafy vegetables, Chillis: green, Ladys finger, Parwal, Patal/Kundru, Cauliflower, Cabbage, Gourd, Pumpkin, Peas, Green, French beans, Barbate, Lemon, Other Vegetables (rural), Other Vegetables (urban)
Fruits	Banana, Jackfruit, Watermelon, Pineapple, Coconut, Coconut: green, Guava, Singara, Orange, Mausami, Papaya, Mango, Kharbooza, Pears, Naspati, Berries, Leechi, Apple, Grapes, Other Fresh Fruits (rural), Other Fresh Fruits (urban)
Meat	Chicken, Goat Meat/Mutton, Beef/ Buffalo Meat, Pork
Eggs	Eggs
Fish and other seafood	Fish, Prawn, Birds, Crab, Oyster, Tortoise etc.
Legumes, nuts and seeds	Arhar, Tur, Gram (split), Gram (whole), Moong, Masur, Urd, Peas, Khesari, Other Pulses, Gram Products, Besan, other pulse products, Baby food, Coconut, Copra, Groundnut, Dates, Cashewnut, Walnut, Other Nuts
Milk and milk products	Milk: liquid, Milk: condensed/ powder, Curd, Ghee, Other Milk Products (rural), Other Milk Products (urban)
Sweets	Sugar – PDS, Sugar – other sources, Gur, Candy, Misri, Honey
Oils and fats	Vanaspati, Margarine, Mustard oil, Groundnut oil, Coconut oil, Refined oil Sunflower, Soyabean, Saffola, etc. Edible oil: Others
Miscellaneous	Ice-cream (rural), Ice-cream (urban), Cold Beverages: bottled/ canned, Fruit Juice and Shake, Other Beverages: Cocoa, Chocolate etc. (rural), Other Beverages: Cocoa, etc. (urban), Cooked Meals purchased, Cooked Meals received as assistance, Cooked Meals received free in workplace, Cooked Snacks purchased [Samosa, Puri, Paratha, Burger, Chowmein, Idli, Dosa, Vada, Chops, Pakoras, Pao bhaji, etc.] (rural), Cooked Snacks purchased [Samosa, Puri, Paratha, Burger, Chowmein, Idli, Dosa, Vada, Chops, Pakoras, Pao bhaji, etc.] (urban), Other served Processed Food (rural), Other Served Processed Food (urban), Cake, Pastry, Prepared Sweets (rural), Cake, Pastry, Prepared Sweets (urban), Biscuits, Chocolates (rural), Biscuits, Chocolates (urban), Papad, Bhujia, Namkeen, Mixture, Chanachur (rural), Papad, Bhujia, Namkeen, Mixture, Chanachur (urban), Chips, Pickles, Sauce, Jam, Jelly, Other packaged processed food (rural), Other packaged processed food (urban), Toddy, Country Liquor, Beer, Foreign/ Refined Liquor or Wine, Raisin, Kishmish, Monacca, etc. Other dry fruits, Ginger, Garlic, Jeera, Dhania, Turmeric, Black Pepper, Dry Chillies, Tamarind, Curry Powder, Oilseeds, Other Spices, Tea: Cups, Tea: Leaf, Coffee: Cups, Coffee: Powder,Pan: Leaf, Pan: Finished, Ingredients for Pan

S3 Table. Food Processing.

Food Category	Food items in the data
Processed and Ready to eat Food	Ice-cream (rural), Ice-cream (urban), Cold Beverages: bottled/canned, Fruit Juice and Shake, Other Beverages: Cocoa, Chocolate etc. (rural), Other Beverages: Cocoa, etc. (urban), Cooked Snacks purchased [Samosa, Puri, Paratha, Burger, Chowmein, Idli, Dosa, Vada, Chops, Pakoras, Pao bhaji, etc.] (rural),Cooked Snacks purchased [Samosa, Puri, Paratha, Burger, Chowmein, Idli,Dosa, Vada, Chops, Pakoras, Pao bhaji, etc.] (urban), Other Served Processed Food (rural), Other Served Processed Food (urban), Cake, Pastry, Prepared Sweets (rural), Cake, Pastry, Prepared Sweets (urban), Biscuits, Chocolates (rural), Biscuits, Chocolates (urban), Papad, Bhujia, Namkeen, Mixture, Chanachur (rural), Papad, Bhujia, Namkeen, Mixture, Chanachur (urban), Chips, Pickles, Sauce, Jam, Jelly, Other packaged processed food (rural),Other packaged processed food (urban) , Cooked Meals Purchased, Cooked Meals received as assistance, Cooked Meals received free in workplace

S4 Table. Cluster Information.

Cluster Name	Size	Size Percentage
P_A	8764	4.88
P_B	12211	6.80
P_C	17191	9.57
S_A	25651	14.29
S_B	11761	6.55
S_C	16935	9.43
M_A	33769	18.81
M_B	34165	19
M_C	2924	1.63
M_D	16108	8.97

S1 Fig. Selection of Indian Food systems. Based on maximum average silhouette score, we select 10 clusters i.e. Indian food systems.

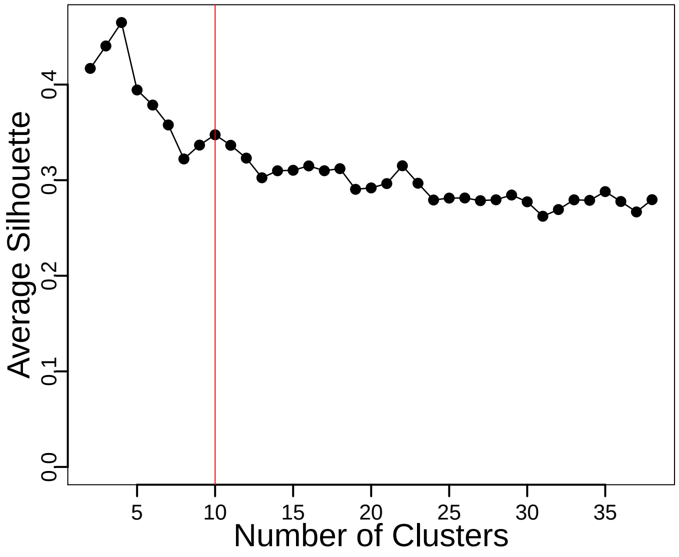

S2 Fig. Three groups of Indian food systems- production-based food systems, subsidy-based food systems, and market-based food systems vary among 15 food system parameters. Production, subsidy and market denotes calorie from home produced food, subsidised food, and market-purchased food. The abbreviations in the figure are- SIDD-Simpsons Index of Dietary Diversity, SIFPD- Simpsons Index of Food Production Diversity, GHG- greenhouse gases, WF- Water footprint, DPCE- Daily per capita income in USD PPP.

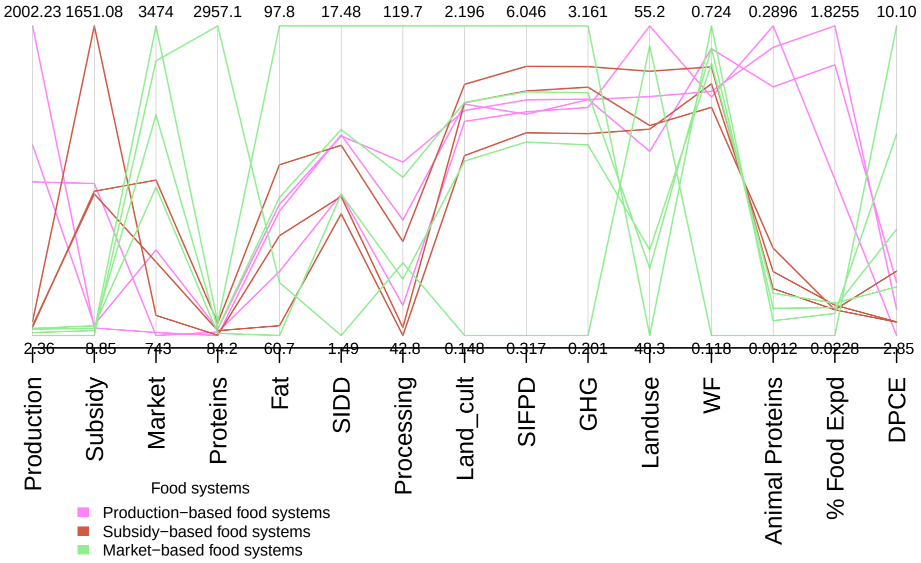

S3 Fig. State and Union Territories map of India.

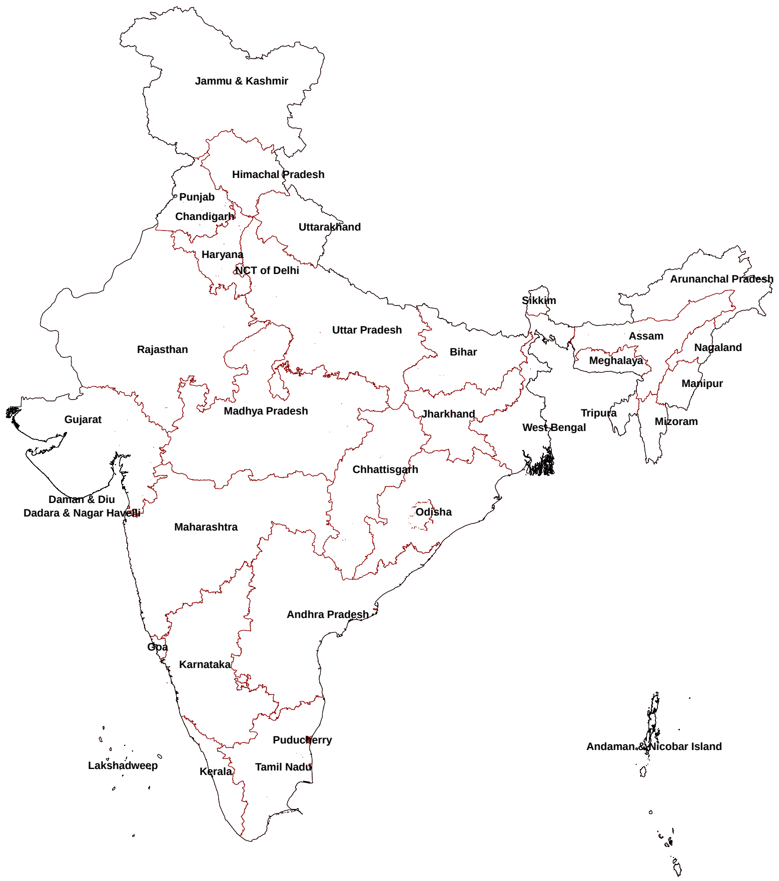

Supplement: S1 Appendix — (PDF) [file pone.0270342.s001.pdf]
