# Supplementary material for: India consists of multiple food systems with scoioeconomic and environmental variations
Source: PLoS One. 2022 Aug 26;17(8):e0270342. doi: 10.1371/journal.pone.0270342 (PMC9416984; doi:10.1371/journal.pone.0270342)
Supplement: Attachment — Submitted filename: Response to reviewers.pdf [file pone.0270342.s002.pdf]

Responses to the comments from the reviewers

Reviewer 1

It is a good piece of work covering a relatively less explored research topic of the "food system" in the Indian context.

Thank you very much for appreciating our manuscript on the topic of Indian Food System and within the scope of PLOS ONE, and providing the constructive comments. These comments helped us to improve our manuscript. We addressed all the comments in the revised version.

1. Consider rewriting the title, it's not conveying the gist of the article with clarity.

Thanks for conveying the concerns about the title. We modify the title as follows:

"India consists of multiple food systems with scoioeconomic and environmental variations"

2. The spatial dimensions in this manuscript is missing; authors have analyzed 0.2 Million samples spread across 8k villages. However, only summarized results are shown; not providing any regional details - not even state wise. I suggest doing some efforts to present results spatially. Authors can try choropleth mapping (Production, Subsidy and Market) to show food system dominance by the district.

Thank you for the suggestion. Following the suggestion, we highlighted the spatial dimension of the ten Indian food systems in the revised manuscript in all the three food systems. Further, we moved our diagram on the spatial distribution of the food systems from the supporting information to the main text. The highlighted descriptions on the spatial dimension are as follows:

"We consider the three food systems P_A, P_B, and P_C production-based because home-produced foods contribute to at least 40% (Fig 2). Among these food systems, P_A and P_B have the highest share of subsidized and purchased food, i.e., around 30% and 50% of the calorie intake, respectively. Spatially, these food systems are prevalent in various parts of India (see Fig 1). The food system P_A is present sporadically across Karnataka, Chhattis-

garh, Odisha, Jammu and Kashmir, Himachal Pradesh, Uttarakhand, and north-eastern states of India. Food system P_B is prevalent to a varying degree in most of India except Kerala, Tamil Nadu, Andhra Pradesh, and most north-eastern states. Food system P_C is predominant across northern, central, and eastern India and Arunachal Pradesh, Nagaland, Manipur, and Assam."

"The subsidy-based food systems are predominant mainly in India's southern and eastern regions (see Fig 1). Additional food subsidies by the state government over and above India's government lead to the prevalence of subsidy-based food systems in these regions [1]. The food system S_A is prevalent in Kerala, Tamil Nadu, Karnataka, Andhra Pradesh, Odisha, Himachal Pradesh, Jammu and Kashmir, Mizoram, Tripura, and Meghalaya. The food system S_B prevails in Chhattisgarh, parts of Tamil Nadu and Odisha, and Maharashtra's eastern districts. The states of Andhra Pradesh, Uttarakhand, Mizoram, Tripura, and Himachal Pradesh consist of the food system S_C."

"Looking at the spatial distribution, the food system M_C is spread thinly and uniformly across India (see Fig 1). Food system M_A is prevalent in Kerala, Maharashtra, Gujarat, Meghalaya, Sikkim, and partly in Karnataka, Jharkhand, Assam West Bengal. The food system M_B is prevailing across Maharashtra, Madhya Pradesh, Rajasthan, Punjab, Jharkhand, Nagaland, and Manipur. Food system M_D is observed mainly in Punjab, Haryana, western Uttar Pradesh, and sporadically in Arunachal Pradesh, Madhya Pradesh, Maharashtra, Jharkhand."

4. Figure 1 (and fig. 3, fig. 5): Worth elaborating or finding the reasons why SIFPD not translating into SIDD especially for production-based food systems (PA), and subsidy-based as well as market-based food systems.

We thank the reviewer for highlighting the issue of SIFPD not translating into SIDD. We elaborate our findings in the result section accordingly. Our elaboration mainly focuses on the production-based food systems because of a huge contribution of home-produced foods in these diets. Nevertheless, we also briefly mentioned SIFPD in the other two food systems and in the discussion sections. For this, we made the following changes:

"Share of calories from production, subsidy and market affects households' dietary diversity (Fig 2). SIDD varies from 0.58 to 0.67 in the production-

based food systems. The food system P_B consumes a more diverse diet than the other two because it has the largest share of calories from the market. Market-purchased foods contribute 743, 1498, and 769 kcal/CU/day in the food system P_A, P_B, and P_C, respectively. Although the food system P_A produces the most diverse food with a SIFPD of 0.28, its dietary diversity is the lowest. SIFPD is 0.23 in food system P_B and 0.26 in food system P_C. Because of these low values, SIFPD did not translate into SIDD in these food systems. In order words, the current food production diversity of the farmers is not diverse enough to enrich their dietary diversity, either due to lower production amounts or growing only a few food groups. Lower dietary diversity among these households could be addressed through interventions like kitchen gardening."

"In the subsidy-based food systems, dietary diversity decreases as the share of subsidized food increases, with SIDD varying from 0.56 to 0.64 (Fig 4). The dominance of cereals in subsidized foods leads to low dietary diversity in these food systems. For example, the food system S_B with a higher subsidized calorie intake has lower dietary diversity than the food system S_A. However, a higher share of calories from market-purchased food increases dietary diversity. These food systems represent 30.23% of Indian households (see S4 Table). Because of a low share of home-produced foods, these food systems also have a low SIFPD, i.e., below 0.1."

"The households in the food system M_C depend on processed and ready to eat foods for 93% of the calories and live in the urban sector (Fig 6 and Fig 7). The other three food systems, M_A, M_B, and M_D, have 5% calories from processed and ready to eat foods. Dietary diversity increases with an increase in income. It varies from 0.64 to 0.72 in the market-based food systems M_A, M_B, and M_D (Fig 6). Almost half of the Indian households (48%) meet their dietary requirements mainly through market purchased foods. These food systems have a negligible share of home-produced foods with low SIFPD, i.e., below 0.05."

"For the first time, we analyze the home-produced foods' role in Indian food systems via contribution to the calories, diversity of food production, and the cultivated land variables. We found that calorie intake increases with a high share of home-produced calories and landholding. However, a high production diversity did not translate into a high dietary diversity. These food systems have an overall low production diversity with SIFPD value below 0.3. Thus, adequate strategies are needed to increase production

diversity, e.g., kitchen gardening, to improve the dietary diversity of Indian households largely dependent on home-produced food. Currently, market purchased food contributes to higher dietary diversity among farm families in India."

5. Similarly worth highlighting the reasons why water and GHG footprints vary significantly among the sub-systems? This would bring more clarity in targeting/promoting the food systems considering

Thank you for highlighting the variation among water and GHG footprints among the sub-systems. Wide variation among calorie intake even among the food sub-systems results in varying environmental footprints in particular GHG emissions. Apart from calorie intake diet composition is main contributor in varying environmental footprints. However, we did not study diet composition, limiting our ability to interpret these varying environmental footprints. Nevertheless, we explain the variations in the comparison of Indian Food Systems section in our revised manuscript. For this, we made the following changes:

"Indian food systems vary widely in environmental footprints, especially among market-based and subsidy-based food systems. These variations mainly result from a large difference in calorie intake within these food systems. Malnutrition co-exists within these food systems with undernourishment (food systems S_A and M_A) and overconsumption (food systems S_C, M_B and M_D). Overall GHG emissions are proportionate to the calorie intake in Indian food systems. However, for water footprints, it is not the case. The composition of diets with variation in amounts of environmental footprint intensive products, e.g., rice and animal-sourced foods, are responsible for varying environmental footprints [2]. A multi-faceted approach to addressing malnutrition, dietary changes, and sustainable food production may help make Indian food systems more sustainable."

"Our study finds a higher environmental footprint in the north Indian states of Punjab, Haryana, and Western Uttar Pradesh. These regions of India have overconsumption, with higher dairy products consumption, resulting in higher environmental footprints [2–4]. Dairy product consumption needs to be reduced in these regions. Reduced dairy products consumption should be supplemented with ruminant numbers reduction to avoid the rebound effect [5]."

“Our analysis limits our explanation of the food systems’ environmental footprints because of not considering dietary composition. Investigation of the dietary composition could better explain the reasons behind variation on these footprints [2]. Instead, we infer findings from Athare and colleagues [2] while interpreting our result, which is also our data source.”

6. Presentation style: The article uses single charting style - only bar graphs. Tables and maps are absent. Summarized results by the state would certainly add value to the article.

Many thanks for the suggestion. We incorporate the suggestion by including a map of spatial distribution of Indian food systems at the state level. However, we do not include any tables as the bar graphs best represent our results. Nevertheless, we have tables in the supplementary information of the manuscript.

Reviewer 2

This study provides a holistic understanding of various food systems of Indian households, namely production, subsidy, market-based food systems. This paper is publishable after addressing the following issues.

Thank you for your comments, we address the issues raised by you in below comments.

The Introduction could relate to the broader discourse on undernutrition, overnutrition, and nutritional deficiencies in the various sectors of Indian society. This is particularly important in the context of increasing non-communicable lifestyle diseases, such as obesity, type 2 diabetes, certain cancers, hypertension, heart diseases, and mental health, that are related to diet and nutrition. The current introduction is very limited in scope to demonstrate the societal value of this research.

We thank reviewer for the suggestion to include the broader discourse on malnutrition. Following the suggestion, we elaborate our introduction on malnutrition as follows:

"At the same time, India is also facing the triple burden of malnourishment [6,7]. Currently, 14% of its population is undernourished, [8] and 19.7% of its adults suffer from overweight and obesity [9]. Increased overweight population and associated noncommunicable diseases have become a public health issue that is widely spread across urban and rural areas [7]. Mainly, overweight and obesity has increased more in women living in rural areas and urban slums compared to non-slum urban areas [6]. Additionally, India has a high share of undernourished population, mainly among adolescent girls, pregnant and lactating women, and children [10] although undernutrition has been rapidly reduced in the country over the last decade [6, 10]. The malnutrition is associated more with food quality than its quantity [7]. Thus, there is a need to understand variation in food systems across India for addressing all forms of malnourishment."

However, we briefly mentioned issues related to noncommunicable diseases because our study only slightly touches the health issues, instead of focusing on it.

A question arises whether the survey reported in this paper is the latest

available survey. It is also helpful to address the limitations of using existing surveys like this. It is not clear from the analysis if this survey also includes data on meat and fish consumption. This paper could benefit from an analysis of increasing meat, fish, and dairy consumption among the emerging middle-class consumers in India.

Thank you for the query. The Household Consumer Expenditure Survey 2011-2012 used in this paper is the latest openly available survey data for food consumption in India. It provides data on various food items, including fish, dairy, egg and meat consumption. We elaborate our description of data addressing these points in the revised manuscript as follows:

"We use the latest 68th round of Household Consumer Expenditure Survey (HCES) 2011–12 for our food system analysis. This dataset provides the contemporary understanding of food systems of Indian households based on various components. It is the latest openly available household survey data for food consumption in India. The survey has information on the consumption of home-produced foods, subsidized foods, and food purchased from the market for different food items, including fish, dairy, egg and meat (see S2 Table)."

Additionally, we also discussed the limitation of this data as it is 10 years old. Mainly, we highlighted a need for recent data to understand the current food systems. Since the data, we are using, provides information for only one year, it is hard for us to analyze changes in diets across India. However, we also mention a need for time-series analysis of household consumption data to understand changing food systems and dietary habits across India in our revised discussion section as follows:

"Since we used HCES 2011–12 data, the food systems, we identified, are around ten years old. Thus, there is a need to use the recent household survey data to understand the current food systems, once it is available, because food systems are changing. Further, analysis of HCES data from different periods would also provide new insights on changes in food systems across India."

The analysis of calorie intake by households with the three food systems could have been more clearer. It is particularly important to explain why those with household production have lower dietary diversity and what policy interventions can make this food system responsive to household prosperity,

human health, and the natural environment.

We thank the reviewer for comment. Following the comment, we explain the reason behind a lower dietary diversity for the food producing household, and also suggest a need for policy interventions to increase their dietary diversity as follows:

“Share of calories from production, subsidy and market affects households’ dietary diversity (Fig 2). SIDD varies from 0.58 to 0.67 in the production-based food systems. The food system P_B consumes a more diverse diet than the other two because it has the largest share of calories from the market. Market-purchased foods contribute 743, 1498, and 769 kcal/CU/day in the food system P_A, P_B, and P_C, respectively. Although the food system P_A produces the most diverse food with a SIFPD of 0.28, its dietary diversity is the lowest. SIFPD is 0.23 in food system P_B and 0.26 in food system P_C. Because of these low values, SIFPD did not translate into SIDD in these food systems. In order words, the current food production diversity of the farmers is not diverse enough to enrich their dietary diversity, either due to lower production amounts or growing only a few food groups. Lower dietary diversity among these households could be addressed through interventions like kitchen gardening.”

“For the first time, we analyze the home-produced foods’ role in Indian food systems via contribution to the calories, diversity of food production, and the cultivated land variables. We found that calorie intake increases with a high share of home-produced calories and landholding. However, a high production diversity did not translate into a high dietary diversity. These food systems have an overall low production diversity with SIFPD value below 0.3. Thus, adequate strategies are needed to increase production diversity, e.g., kitchen gardening, to improve the dietary diversity of Indian households largely dependent on home-produced food. Currently, market purchased food contributes to higher dietary diversity among farm families in India.”

Last but not least, the authors may like to strengthen the conclusion and policy recommendations. Revision of the Introduction along the line suggested above will be helpful to rewrite the Conclusion.

We follow your suggestion and revise the introduction, limitation, conclusion and policy recommendation section of the paper. Our revised policy

recommendations read as follows:

"Our study of Indian food systems could help better target policies according to different food systems as tailored interventions to address malnutrition, dietary diversity, and environmental sustainability issues. Production-based food systems face undernourishment, whereas home-produced food is not contributing to dietary diversity. Policy interventions like kitchen gardening among farm families may help increase dietary diversity, which is currently low. Kitchen gardening could include the production of seasonal vegetables and perennial fruits to meet the nutritional needs of the households. Additionally, these households need support to increase their agricultural productivity and off-farm incomes to supplement home-produced food when it is not enough to nourish throughout the year. Interventions to increase food production need to be carefully designed to have synergistic effects on social, economic, and environmental systems, tackling the current sustainability trade-offs of food systems [11]. Subsidy-based food systems face undernourishment and overconsumption with lower dietary diversity. Here, policies on better-targeting food subsidies, dietary awareness, and diversifying food subsidies from cereals will help address malnutrition and dietary diversity issues. Focused policies on healthy diet awareness in middle-class families would help address overconsumption in market-based food systems. Here, undernourishment needs to be addressed by tackling issues related to urban poverty. Reducing overconsumption and lower animal protein intake will transform Indian food systems into healthy and sustainable ones."

References

[1] Desai S, Vanneman R. Enhancing Nutrition Security via India's National Food Security Act: Using an Axe instead of a Scalpel? In: India Policy Forum:[papers]. India Policy Forum. Conference. vol. 11. NIH Public Access; 2015. p. 67.

[2] Athare TR, Pradhan P, Kropp JP. Environmental implications and socioeconomic characterisation of Indian diets. Science of The Total Environment. 2020; p. 139881.

[3] Aleksandrowicz L, Green R, Joy EJM, Harris F, Hillier J, Vetter SH, et al. Environmental impacts of dietary shifts in India: A modelling

study using nationally-representative data. Environment International. 2019;126:207–215. doi:10.1016/j.envint.2019.02.004.

[4] Rao ND, Min J, DeFries R, Ghosh-Jerath S, Valin H, Fanzo J. Healthy, affordable and climate-friendly diets in India. Global Environmental Change. 2018;49:154–165. doi:10.1016/j.gloenvcha.2018.02.013.

[5] Bodirsky BL, Pradhan P, Springmann M. Reducing ruminant numbers and consumption of animal source foods are aligned with environmental and public health demands. J Sustainable Organic Agric Syst. 2019;69(1):25–30.

[6] Nguyen PH, Scott S, Headey D, Singh N, Tran LM, Menon P, et al. The double burden of malnutrition in India: Trends and inequalities (2006–2016). Plos one. 2021;16(2):e0247856.

[7] Meenakshi J. Trends and patterns in the triple burden of malnutrition in India. Agricultural Economics. 2016;47(S1):115–134.

[8] FAO, IFAD, UNICEF, WFP, WHO. The State of Food Security and Nutrition in the World 2020. Transforming food systems for affordable healthy diets. https://doi.org/10.4060/ca9692en: FAO, Rome.; 2020.

[9] WHO. Global health observatory data. World Health Organization : Geneva. 2019;.

[10] Narayan J, John D, Ramadas N. Malnutrition in India: status and government initiatives. Journal of public health policy. 2019;40(1):126–141.

[11] Warchold A, Pradhan P, Thapa P, Putra MPIF, Kropp JP. Building a unified Sustainable Development Goals (SDGs) database: Why does SDG data selection matter? Sustainable Development. 2022;doi:10.1002/sd.2316.
